# What Kills the Patient—Peritonitis or Infection?

**Published:** 1894-07

**Authors:** Byron Robinson

**Affiliations:** 34 Washington Street; Chicago, Ill.


### WHAT KILLS THE PATIENT—PERITONITIS OR INFECTION?

BY BYRON ROBINSON, Chicago, Ill.

IN CONVERSATION with a gynecologist the other day, he remarked that his patient died on the fifth day from peritonitis. A curious view prevails among physicians that peritonitis causes death; that if peritonitis can only be avoided the patient is safe. The dread of many a physician is peritonitis; as if the patient hung on a thread and peritonitis would snap it ruthlessly asunder. A few careful autopsies on patients who have died from peritonitis would soon dispel this illusion. One sees bands, adhesions and barriers of exudate here and there. Partitions and departments have been formed in the abdominal cavity. Pools of pus have been corralled and circumscribed. Viscid, jelly-like substances surround ill-appearing localities. The most angry complaining viscera have become the most imbedded and fixed. Yet the physician says the patient died of peritonitis. What a grand mistake. The peritonitis made wonderful efforts to save the patient's life, or had it not been for the peritonitis the patient would have long before passed to the tents on the greensward whose curtains never swing backward.

An example will suffice to illustrate that peritonitis attempts to save life instead of destroy it:

Mrs. A. came to the hospital with hernia strangulated for three days. Pulse 110, and shortly after the operation temperature 103. The operation removed a gangrenous gut strangulated by Poupart's and Gimbernat's ligaments. The gut was reduced so far as Poupart's ligament was concerned, but the strangulation was not relieved for a second constriction tightly embraced the gut-loop from the chronic imflammation, which had existed for three years previous. Abdominal section was made and the loop in intestine forcibly pulled out from the inside of the peritoneal sac. Six inches of the blackened bowel resected and a Murphy's button inserted. The peritoneal redness, the vascular congestion, the deranged pulse and temperature, showed that she had peritonitis. The lower half of the peritoneum presented the suffused tint of a setting sun. The patient quickly rallied. The temperature became normal, the pulse became 80 and she was hungry. All was going well until about the seventieth hour, when a sudden disturbance arose and she became pale. The temperature fell to 98, the pulse flew up to 140. She became restless. Tympanitis rapidly arose and she died about ninety hours after the operation. The autopsy revealed a perforation at the side of the button on the distal side of the mesentery.

The autopsy, performed by Dr. Brown, of the Post-Graduate School, revealed only a few square inches of what one would call peritonitis, but several square inches of dull, dark peritoneum around the seat of perforation. No adhesions were formed. What killed this patient—peritonitis or infection? It is easy to answer that infection was the head and front in the cause of death. If peritonitis could have arisen, the woman's life might have been saved by barriers of exudate. Inflammation is the mode that the peritoneum takes to preserve life. Peritonitis is the standing army against the invasion of infection, and the omentum is like a man-of-war, ready at a moment's notice to go to the points of peritoneal infection to capture the invader. Observe, for a moment, the three regions of the peritoneum where infection invades—namely, between the colon and diaphragm, around the appendix and in the pelvis,—and note how adhesions promptly check invasions.

Few autopsies are done without peritonitic adhesions being found in the pelvis around the cecum or in the region of the gall-ducts. In such cases, life was absolutely saved by inflammation, by peritonitis throwing out barriers of exudates,—advance guards against foreign invasion of merciless soldiers, whose sole purpose is destruction and death. Now, turn attention to the small intestines, around which are found but few exudates or adhesions, for the simple reason that death occurs before peritonitis can form barriers to stop death on its way, which is simply infection (the colon bacillus). If the peritoneum on the small intestines had time to inflame, it would throw out exudates to surround and capture the invader. Peritonitis would save life by starving out the infectious invader by coralling him in a limited space, *e. g.*, a pool of pus, whence sterility of the pathogenic microbe arises. Modern research asserts that microbes are the cause of peritonitis, but the adhesions, bands and exudates show how hard the peritoneum tries to stop the advances of such enemies of mankind. If it were not for peritonitis death would soon end the scene. But, while blood corpuscles swarm to the seat of invasion and quickly attempt to surround the infectious invader by burying him with the dead of the battle, infection is only conquered by being buried in the *débris* of its own devastation. As a proof that peritonitis saves life, observe that the most favorable cases of abdominal section are those where collections of pus are circumscribed. The infection has been checked by peritonitis, *e. g.*, in the pelvis, around the cecum, or gall-bladder. The circumscribed invader has been starved out by cutting off supplies of food or space. Hence, infection is the dreaded foe, while peritonitis is the savior of life.

Peritonitis builds solid forts around the ends of the Fallopian tubes, the appendix and the gall-bladder, which alone insure liberty of life to the viscera. We are just beginning to interpret the wonderful workings of the peritoneum. I was once infected in my finger, and my whole arm readily served as a test-tube of culture medium in a few hours. There was no peritoneum to protest by inflaming against the invader, and I was soon delirious. Finally, the white blood-corpuscles arose in sufficient numbers to overcome the intruders and starve them out. It required some six weeks to rout the infectious invader. A peritoneum would have likely coralled the infection by an exudate in a few days. Peritonitis tends to save life. Infection tends to destroy it.

34 Washington Street.

---

The Marriage of Syphilitics.—In general the advice to syphilitics would be not to marry, but Dr. William G. Porter believes in doing the syphilitic justice, and for this reason he looks at the subject with very lenient eyes. He divides the disease into the benignant, the moderate and the malignant variety. The stages explain themselves. The first form recovers with no treatment, while the second and third need a skilful physician's advice, and after all disappearance of the symptoms the patient may safely marry in two years.—*Maryland Medical Journal.*

